# Comparison of statistical algorithms for daily syndromic surveillance aberration detection

**DOI:** 10.1093/bioinformatics/bty997

**Published:** 2019-01-25

**Authors:** Angela Noufaily, Roger A Morbey, Felipe J Colón-González, Alex J Elliot, Gillian E Smith, Iain R Lake, Noel McCarthy

**Affiliations:** 1 Statistics and Epidemiology, Warwick Medical School, University of Warwick, Coventry, UK; 2 Real-time Syndromic Surveillance Team, National Infection Service, Public Health England, Birmingham, UK; 3 School of Environmental Sciences, University of East Anglia, Norwich, UK; 4 Population Evidence and Technologies, Warwick Medical School, University of Warwick, Coventry, UK

## Abstract

**Motivation:**

Public health authorities can provide more effective and timely interventions to protect populations during health events if they have effective multi-purpose surveillance systems. These systems rely on aberration detection algorithms to identify potential threats within large datasets. Ensuring the algorithms are sensitive, specific and timely is crucial for protecting public health. Here, we evaluate the performance of three detection algorithms extensively used for syndromic surveillance: the ‘rising activity, multilevel mixed effects, indicator emphasis’ (RAMMIE) method and the improved quasi-Poisson regression-based method known as ‘Farrington Flexible’ both currently used at Public Health England, and the ‘Early Aberration Reporting System’ (EARS) method used at the US Centre for Disease Control and Prevention. We model the wide range of data structures encountered within the daily syndromic surveillance systems used by PHE. We undertake extensive simulations to identify which algorithms work best across different types of syndromes and different outbreak sizes. We evaluate RAMMIE for the first time since its introduction. Performance metrics were computed and compared in the presence of a range of simulated outbreak types that were added to baseline data.

**Results:**

We conclude that amongst the algorithm variants that have a high specificity (i.e. >90%), Farrington Flexible has the highest sensitivity and specificity, whereas RAMMIE has the highest probability of outbreak detection and is the most timely, typically detecting outbreaks 2–3 days earlier.

**Availability and implementation:**

R codes developed for this project are available through https://github.com/FelipeJColon/AlgorithmComparison

**Supplementary information:**

Supplementary data are available at *Bioinformatics* online.

## 1 Introduction

Epidemiological surveillance is becoming more important due to the increasing public health threats resulting from the quick spread of infections, especially as the world population increases and environmental risks augment. Public health authorities seek efficient algorithms that can detect unusual increases in infections quickly, so that they can investigate the sources of spread and ultimately take control measures. A main challenge for such algorithms is that they must be completely automated and must work across a range of different infections and syndromes encountered in real life in order to be useful in daily practice.

Most of the existing literature (Bédubourg and Stratt, 2017; Buckeridge and Burkom, 2010; Enki *et al.*, 2016; Spreco *et al.*, 2017; Texier *et al.*, 2017; Unkel *et al.*, 2012; Yang *et al.*, 2018) considers and evaluates surveillance algorithms for weekly data. There is, however, a rising interest in daily surveillance (e.g. Abat *et al.*, 2016; Mathes *et al.*, 2017; Morbey *et al.*, 2015; Smith *et al.*, 2016; Vial *et al.*, 2016). One of the initial motivations of daily surveillance was the early warning of bio-terrorist incidences. However, nowadays, daily surveillance is seen as important for various purposes such as situation awareness during events, reassurance about lack of incidents during mass gatherings and providing earlier detection for quicker control. Syndromic surveillance is generally performed daily based on diagnostic symptoms like cough, fever or diarrhoea, which are available before a laboratory-confirmed causal pathogen has been identified. Syndromic Surveillance has been in routine use at Public Health England (PHE) and its predecessors the Health Protection Agency and the Public Health Laboratory Service since 2001. Within PHE, syndromic surveillance is coordinated by the Real-time Syndromic Surveillance Team (ReSST). ReSST currently monitors general practitioner (GP) consultations using an in-hours syndromic system (GPIHSS) (Harcourt *et al.*, 2012a,b) and an out-of-hours and unscheduled care system (GPOOHSS) (Harcourt *et al.*, 2012a,b), calls to a national telephone health service (NHS 111) (Harcourt *et al.*, 2016) and emergency department attendances (EDSSS) (Elliot *et al.*, 2012). 

The aim of this paper is to investigate the performance of three extensively used multi-purpose outbreak detection algorithms in monitoring daily syndromic data across a range of scenarios representing real-life syndromic activity. We base these scenarios on PHE’s syndromic surveillance system and the various syndromic data signals it encounters. We, therefore, compare: the multi-level regression approach known as the ‘rising activity, multilevel mixed effects, indicator emphasis’ (RAMMIE) method (Morbey *et al.*, 2015) developed and currently used at PHE for syndromic daily surveillance; the improved quasi-Poisson regression-based (Noufaily *et al.*, 2013) method (also known and referred to in this paper as Farrington Flexible) developed and currently used in PHE for weekly detection of infectious disease outbreaks; the ‘Early Aberration Reporting System’ (EARS) method (Hutwagner *et al.*, 2003) based on Shewhart control charts, developed and used as the standard system (since 11 September 2001) at the United States CDC for conducting weekly syndromic surveillance (Fricker *et al.*, 2008). In doing so, the study addresses key challenges in epidemiological surveillance. It provides a multi-purpose setting for evaluating algorithms based on simulations representing the range of real-life syndromes. It also addresses the challenge of monitoring daily counts for potential alarms, a theme that has not been thoroughly explored in the literature as the main focus has been on weekly surveillance. In addition, it presents the first formal evaluation of RAMMIE since its introduction in 2013.

Farrington Flexible and EARS are methods usually applied to weekly data, therefore we adapted them for daily surveillance by using 7-day moving totals. We tested all four variants of the EARS method (called C1, C2, C3 and NB). Also, we developed a modified version of RAMMIE which includes testing for long-term trends. Our comparison thus involves the two major surveillance algorithms (regression-based RAMMIE and Farrington Flexible) used at PHE and arguably the most commonly used surveillance algorithm, EARS, which includes non-regression-based variants, and so we provide a variety of approaches to contrast. The main challenge facing such algorithms is to control the false alarms whilst keeping a good power of detection, i.e. producing high sensitivity and specificity at the same time. We evaluate the performance of RAMMIE, Farrington Flexible and EARS by comparing the power of detection, sensitivity, specificity and timeliness using extensive simulations based on various scenarios that reflect the range of different data structures encountered in PHE’s syndromic surveillance system and seen in the real world including volume, trend, seasonality and day-of-the-week effects.

In Section 2, we describe the RAMMIE, Farrington Flexible and EARS algorithms. Section 3 explains the simulation study design used to compare the three algorithms and Section 4 introduces the measures used for this evaluation. Section 5 displays the results. We conclude with a final discussion and interpretation of the results in Section 6.

## 2 The algorithms

In this section, we provide a description of the algorithms we compared. These algorithms offer public health bodies a first indication of unusual activity or aberrations in the form of statistical alarms. Within PHE, a risk assessment process follows the identification of statistical alarms after which a smaller proportion of the alarms will be acted upon as required (Smith *et al.*, 2016).

### 2.1 The ‘rising activity, multilevel mixed effects, indicator emphasis’ (RAMMIE) method

RAMMIE (Morbey *et al.*, 2015) fits a multilevel mixed effects negative binomial regression model to historical daily syndromic data counts and provides estimates for current counts at a local, regional and national level in England. The model controls for the days of the week/month and bank holidays which can impact on health care consulting behaviour. Upper prediction intervals for the estimates are used to create thresholds and generate statistical alarms whenever actual counts exceed the thresholds. ReSST uses RAMMIE as the first stage in its risk assessment process for decision-making (Smith *et al.*, 2016). For this study, data was not stratified into different geographies so the RAMMIE models are not multilevel, instead a simplified negative binomial model was used.

RAMMIE uses a denominator as an offset in its regression models to allow for potential large daily fluctuations in daily coverage from data providers. For this study, where coverage does not vary, an offset *N_t_* at day *t* is defined as:

(1) $$\begin{array}{cc} N_{t}=100\{(H_{t}\frac{\sum_{i=1}^{n}\left(y_{i}H_{i}\right)}{\sum_{i=1}^{n}H_{i}}) & +(Sa_{t}\frac{\sum_{i=1}^{n}\left(y_{i}Sa_{i}\right)}{\sum_{i=1}^{n}Sa_{i}})\\ & +(Su_{t}\frac{\sum_{i=1}^{n}\left(y_{i}Su_{i}\right)}{\sum_{i=1}^{n}Su_{i}})\\ & +(W_{t}\frac{\sum_{i=1}^{n}\left(y_{i}W_{i}\right)}{\sum_{i=1}^{n}W_{i}})\}, \end{array}$$

where *H*, *Sa*, *Su* and *W* are binomial variables being either 0 or 1 when day *t* is a public holiday, Saturday, Sunday or other day respectively; *y_t_* is the count on day *t* and *n* the number of days in the baseline dataset.

A negative binomial regression model is fitted to all available baseline data using the following loglinear model which includes the offset, month of the year, day of the week and whether or not a day is a public holiday as independent variables:

(2) $$\ln(y_{t})=\ln(N_{t})+\sum_{i=1}^{7}\beta_{i}D_{it}+\sum_{j=1}^{12}\alpha_{j}M_{jt}+\gamma H_{t},$$

where *D_it_* are seven binary variables for the day of the week, on any particular day *t*, six of these will be zero and the other variable equal to one. *M_jt_* are 12 weighted variables for the months of the year.

An alarm is signalled if the current expected count is larger than three and at the same time higher than the current observed count plus three times the standard deviation; details can be found in Morbey *et al.* (2015).

RAMMIE does not include an independent variable for trends. However, for this study, a modified version of RAMMIE was also created with an added simple linear trend to account for potential long-term trends in the data. This version provided better detection and allowed a fairer comparison with the other algorithms.

### 2.2 The quasi-Poisson regression-based exceedance algorithm

Farrington Flexible (Noufaily *et al.*, 2013) fits a quasi-Poisson regression-based model to weekly confirmed organism counts (by date of report), with mean (expected count) *μ_i_* and variance $\phi \mu_{i}$ at week *t_i_*. To estimate the organism at the current week, the model is fitted to the most recent years (usually 5 years) and includes a linear trend as well as a yearly 10-level factor whose reference period comprises comparable weeks in previous years. The corresponding log-linear model is:

(3) $$\text{log}\;\mu_{i}=\theta +\beta t_{i}+\delta_{j(t_{i})},$$

where $j(t_{i})$ is the seasonal factor level for week *t_i_*, with $j(t_{0})=0$ and $\delta_{0}=0$. In this model, a trend is always fitted, irrespective of its statistical significance, except for special cases where data is very sparse.

A particular week is flagged as being a possible outbreak based on the value of what is known as the exceedance score:

(4) $$X=\frac{y_{0}-{\hat{\mu }}_{0}}{U-{\hat{\mu }}_{0}},$$

where *y*_0_ is the current observed count and ${\hat{\mu }}_{0}$ = exp($\hat{\theta }$ + $\hat{\beta }t_{0}$ + $\delta_{j(t_{0})}$) is the current expected count, $\hat{\theta }$ and $\hat{\beta }$ being the respective estimates of *θ* and *β* from Equation (3). *U*, the upper threshold, is the 100(1-*α*)% negative binomial quantile, *α* being the type *I* error. Another suggested approach to compute *U* uses the 2/3 power transformation of the Poisson distribution which is approximately normal. An alarm is flagged for organism weeks where X≥1. The exceedance score is conditioned to 0 for particular cases that represent high data sparsity.

To reduce the effect of baseline outbreaks on current predictions, the algorithm reweights baseline data. As explained in Noufaily *et al.* (2013), the baseline at week *t_i_* is down-weighted by a factor of the Anscombe residual when the latter is greater than 2.58 at that week.

This algorithm is implemented in R Development Core Team (2018) and is available via the function Farrington Flexible within the package surveillance (Höhle, 2007; Salmon *et al.*, 2016).

### 2.3 The Early Aberration Reporting System (EARS)

EARS (Hutwagner *et al.*, 2003) is available through its four variants (EARS-C1, EARS-C2, EARS-C3 and EARS-NB), mainly used for monitoring weekly syndromic counts. These methods are particularly useful when limited baseline data is available for undertaking syndromic surveillance. Although the first three variants are labelled *C* after CUSUM, most of them are actually Shewhart range methods using a moving sample average and sample standard deviation (Fricker *et al.*, 2008). For each of the variants EARS-C1, EARS-C2 and EARS-C3, a statistical alarm is produced at week *t* with observed count *Y*(*t*) if statistics *C*_1_, *C*_2_ and *C*_3_ (given below) respectively exceed the baseline count mean plus a multiple of the standard deviation:

(5) $$C_{1}(t)=\frac{Y(t)-\mu_{1}(t)}{\sigma_{1}(t)},$$

where $\mu_{1}(t)=\frac{1}{7}\sum_{i=t-1}^{t-7}Y(i)$ and $\sigma_{1}^{2}(t)=\frac{1}{6}\sum_{i=t-1}^{t-7}(Y(i)-\mu_{1}(i){)}^{2}$ are respectively the moving sample mean and the moving sample standard deviation.

(6) $$C_{2}(t)=\frac{Y(t)-\mu_{2}(t)}{\sigma_{2}(t)},$$

where $\mu_{2}(t)=\frac{1}{7}\sum_{i=t-3}^{t-9}Y(i)$ and $\sigma_{2}^{2}(t)=\frac{1}{6}\sum_{i=t-3}^{t-9}(Y(i)-\mu_{2}(i){)}^{2}$ are respectively the moving sample mean and the moving sample standard deviation.

(7) $$C_{3}(t)=\sum_{i=t}^{t-2}\max[0,C_{2}(i)-1]$$

Alarms for the different variants are produced when corresponding statistics *C*_1_ or *C*_2_ exceed three sample standard deviations above the sample mean or if *C*_3_ exceeds two sample standard deviations above the sample mean.

EARS-NB implements Shewhart regression Poisson and negative binomial charts based on the generalized likelihood ratio statistic and is described in Höhle and Paul (2008). The method is implemented via the algo.glrnb function within the R Development Core Team (2018) surveillance package (Höhle, 2007; Salmon *et al.*, 2016).

## 3 Simulation study

We first describe how baseline data is simulated and second how outbreaks are generated. The simulations reflect the real world experience of the syndromic surveillance systems. A novel and key feature in this paper is the way simulations take into account the day-of-the-week effects based on health-seeking behaviour. These effects are also applied to the outbreaks before combining them with the synthetic baselines (Buckingham-Jeffery *et al.*, 2017). The simulations are a potential resource for similar evaluations and can be used by researchers for testing other algorithms in a daily setting.

### 3.1 Simulated baseline data

The simulations are set to reflect the various syndromes encountered at PHE as well as the reporting patterns. The four services reporting to PHE (i.e. GPOOHSS, GPIHSS, NHS 111 and EDSSS) report data based on the days of the week they operate. GPOOHSS and NHS 111 operate on a 7-day-week basis, with a lower volume of reports during the week and almost double that volume on weekends. GPIHSS operates on a 5-day-week basis (only during weekdays) and portrays two peaks around Mondays and Fridays (the Friday one being smaller). In contrast, for EDSSS the day of the week effects are much smaller.

Simulations are designed to mimic the various syndromes’ properties, including volume, trend, seasonality and weekly patterns. Based on Noufaily *et al.* (2013), data is generated using a negative binomial model (of mean *μ* and variance ϕμ) with dispersion parameter ϕ≥1. We adapt the Noufaily *et al.* (2013) model to incorporate the day-of-the-week effects. Hence, two simulation models are designed, one for each of the 5-day-week and 7-day-week systems. On day *t*, mean μ(t) is defined as:

(8) $$\begin{array}{c} \mu (t)=\text{exp}\;\{\theta +\beta (t+s)+\sum_{j=1}^{k_{1}}\{\gamma_{1}\;\text{cos}(\frac{2\pi j(t+s)}{52\times d})\\ +\gamma_{2}\;\text{sin}(\frac{2\pi j(t+s)}{52\times d})\}\\ +\sum_{j=1}^{k_{2}}\{\gamma_{3}\;\text{cos}(\frac{2\pi j(t+s)}{d})\\ +\gamma_{4}\;\text{sin}(\frac{2\pi j(t+s)}{d})\}\}, \end{array}$$

where *d* is 5(7) for the 5(7)-day-week system. The value $k_{1}=0$ corresponds to no seasonality, $k_{1}=1$ and $k_{1}=2$ to annual and biannual seasonality respectively, while $k_{2}=0$ corresponds to no specific weekly pattern, $k_{2}=1$ and $k_{2}=2$ to one and two weekly peaks respectively. In our simulations, we have considered the real-world variability seen in our systems and characterized it into 16 data scenarios (there is no particular significance for the choice of a total of 16) representing the range of over 12 000 syndromic surveillance time series that PHE analyses daily, taking into consideration different linear trends (*β*), seasonal trends (*γ*_1_ and *γ*_2_), day-of-the-week effects (*γ*_3_ and *γ*_4_), baseline frequencies of reports (*θ*) and dispersions (ϕ). A horizontal shifting parameter (*s*) allows easier control over dates of peaks. Table 1 shows the parameters used to simulate 16 different data scenarios representing most syndromic data signals encountered in England. Table 2 displays examples of 16 syndromes that can show a similar type of behaviour to the 16 simulated scenarios, along with their characterizations. We did not select these 16 syndromes because they were the most clinically important or most likely to have outbreaks. Instead, these syndromes together cover the range of different structures of the different time series monitored daily by PHE.

**Table 1.** Parameters and criteria used to generate the 16 representative signals

Signal	*θ*	*β*	*γ*_1_	*γ*_2_	*γ*_3_	*γ*_4_	ϕ	*s*	*k*_1_	*k*_2_	Trend
1	6	0	0.2	0.2	0.5	0.4	2	29	1	2	0
2	0.5	0	1.5	1.4	0.5	0.4	1	−167	1	2	0
3	5.5	0	0	0	0.3	0.25	1	1	0	2	0
4	2	0	0	0	0.3	0.25	1	1	0	2	0
5	6	0	0.3	2	0.3	0.5	1.5	−50	1	2	0
6	1	0	0.1	2	0.05	0.05	1	−50	1	1	0
7	6	0.0001	0	0	0.6	0.9	1.5	0	0	1	1
8	3	0	1.5	0.1	0.2	0.3	1	−150	1	1	0
9	3	0	0.2	0.1	0.05	0.15	1	−200	1	1	0
10	5	0	0.2	0.1	0.05	0.1	1	0	1	1	0
11	0.5	0	0.4	0	0.05	0.15	1	0	2	1	0
12	9	0	0.5	0.2	0.2	0.5	1	0	1	1	0
13	2	0.0005	0.8	0.8	0.8	0.4	4	57	1	2	1
14	0.05	0	0.01	0.01	1.8	0.1	1	−85	4	1	0
15	3	0	0.8	0.6	0.8	0.4	4	29	1	2	0
16	6	0	0	0	0.8	0.4	4	1	0	2	0

**Table 2.** Characteristics of 16 syndromes representative of the 16 simulated data signals

Signal ID	Related system	Related syndrome	Mean daily count	Yearly variation	5/7 day service	Trend
1	NHS111	Diarrhoea	>100	Moderate	7	No
2	ED	Arthropod bites	<10	Large summer peak	7	No
3	ED	Cardiac	<500	Small	7	No
4	ED	Cardiac admissions (HCU/ICU)	<10	Small	7	No
5	GPIHSS	Allergic rhinitis	>100	Large peak with variable timing	5	No
6	GPIHSS	Heat stroke	<10	Large peak variable with timing	5	No
7	GPIHSS	Herpes zoster	>100	Small	5	Yes
8	GPIHSS	Insect bite	10–100	Large summer peak	5	No
9	GPIHSS	Pertussis	10–100	Moderate	5	No
10	GPIHSS	Pneumonia	>100	Moderate	5	No
11	GPIHSS	Rubella	<10	Moderate	5	No
12	GPIHSS	Upper tract respiratory infection	>100	Moderate	5	No
13	GPOOHSS	Bronchitis	10–100	Moderate	7	Yes
14	GPOOHSS	Hepatitis	<10	Moderate	7	No
15	GPOOHSS	Influenza-like illness	10–100	Large peak with variable timing	7	No
16	GPOOHSS	Urinary tract infection	>100	Small	7	No

**Fig. 1.**
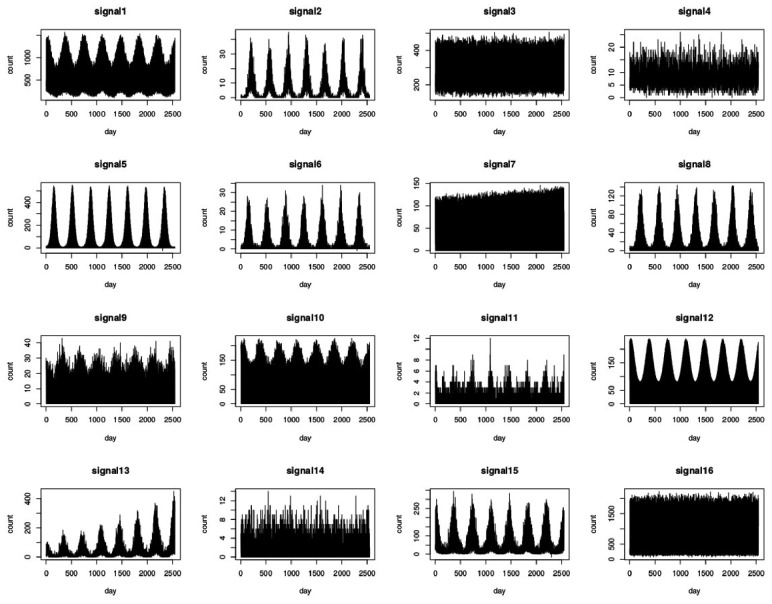
Plots of the 16 simulated data signals

Baseline data, in the absence of outbreaks, is generated using 100 simulations from each of the 16 scenarios; each simulation of size 2548 days (i.e. 7 years consisting of 364 days each or equivalently 52 weeks). Day-of-the-week effects are also reflected within each week. In a 7-day-week system, weekends are set to have around double the volume of reports than weekdays. In a 5-day-week system, weekends are set to zero, whereas weekdays generally consist of 2 peaks, one at the beginning of the week (around Monday) and another later in the week (around Friday). Figure 1 shows the resulting data series and Supplementary Figure S1 shows the first 3 weeks of signals 3 (7-day-week system) and 7 (5-day-week system) to demonstrate the modelled weekly patterns. The outbreaks are added to the most recent 49 weeks (343 days) of the simulated syndromic data.

### 3.2 Simulated outbreaks and public holiday effects

The simulation models described in this paper enable the control of baselines for different scenarios, and therefore outbreaks and unusual increases of different shapes can be added to the baselines. We consider two types of outbreaks: ‘spiked outbreaks’ which last around 3 weeks on average and ‘seasonal outbreaks’ which have a duration of about 8 weeks on average. We also consider the effects of public holidays (called bank holidays in the UK), which usually last 1 or 2 days, because of their impact on syndromic baselines. All outbreaks and public holiday effects take into account the day-of-the-week pattern.

#### 3.2.1 Seasonal outbreaks

Examples of syndromes with seasonal outbreaks are syndromes designed to detect seasonal influenza and allergic rhinitis (i.e. hay fever). Although, baseline data already take into account seasonality, ‘seasonal outbreaks’ differ from the usual seasonality in the sense that the size and timing of their peak is more variable. They are added to a similar window of weeks within each year of simulated data. Based on Noufaily *et al.* (2013), outbreak sizes are simulated using a Poisson distribution with mean equal to *m* times the standard deviation of the baseline count of the day at which the outbreak started and then distributed randomly in time according to a lognormal distribution with mean 0 and standard deviation 0.5. Outbreaks are then re-weighted based on the day of the week they fall on, since outbreaks tend to be influenced by the weekly patterns. In the 5-day-week system, Monday (Tuesday) outbreaks are over-weighted by a factor of 1.5 (1.1). Outbreak days falling on the remaining weekdays are kept the same. In the 7-day-week systems, weekend outbreaks are over-weighted by a factor of 2, whereas weekday outbreaks are kept the same. ‘Seasonal outbreaks’ are only added to signals 5 (*m *=* *1680), 6 (*m *=* *1050) and 15 (*m *=* *3150) as shown in Supplementary Figure S2.

#### 3.2.2 Spiked outbreaks

‘Spiked outbreaks’ are added to the most recent 49 weeks of our 16 simulated signals. They are generated in a similar fashion to ‘seasonal outbreaks’, however they are of shorter duration. Day-of-the-week re-weighting is also considered. ‘Seasonal outbreaks’ were added first to signals 5, 6 and 15, then ‘spiked outbreaks’ were added to all signals. ‘Spiked outbreaks’ of different sizes—very small, small, medium and large using respective *m* values of 2, 3, 5 and 10—have been considered and this study will involve repeating the analysis of the 16 signals 4 times, each time using a different ‘spiked outbreak’ size. For the purpose of demonstration, Supplementary Figure S3 shows baseline data for signals 1, 2, 9 and 12 with examples of medium (*m *=* *5) ‘spiked outbreaks’.

#### 3.2.3 Public holidays

Public holiday effects are added to the simulations following the addition of ‘seasonal outbreaks’ and ‘spiked outbreaks’. We chose the public holiday dates to be on similar days as the United Kingdom ‘bank holidays’ (Supplementary Data). In the 5-day-system, the public holiday count was set to zero and the weekday after the public holiday was multiplied by 1.5; in the 7-day-system, the public holiday count was doubled.

## 4 Evaluation measures

We used different measures to evaluate the performance of the detection systems in the presence of outbreaks. The measures are the power of detection (POD), sensitivity (also known as the true positive), specificity (also known as the true negative or as ‘1-false positive rate’), positive predictive value (also known as PPV or precision) and timeliness. POD is the probability of having an alarm at least once during a spiked outbreak i.e. the probability of detecting the outbreak; sensitivity is the proportion of alarms among spike outbreak days; specificity is the proportion of no alarms among non-outbreak days; PPV is the proportion of detected outbreaks that are true positives i.e. the proportion of detections that are correct; timeliness is the proportion of days elapsed to detect an outbreak since its start. This measure of timeliness prevents undue weight being given to poor performance during a very long outbreak, which is a problem if timeliness is measured as the number of days since the start of an outbreak. If an outbreak was not detected, then timeliness was set to 1. Sensitivity and specificity are a rate per day whereas POD, PPV and timeliness are a rate per outbreak. For each of the 16 simulated signals, all five measures are computed from running the algorithms to the most recent 49 weeks (343 days) of the 100 simulations across each of the four sizes of ‘spiked outbreaks’. We note that we use these measures to evaluate the detection of just ‘spiked outbreaks’ in the presence of ‘spiked outbreaks’, ‘seasonal outbreaks’ and public holidays. Below are the explicit formulae used to compute each measure:

(9) $$\text{POD}=\frac{\text{number}\;\;\text{of}\;\;\;`\text{spiked}\;\;\text{outbreak}s^{\prime }\;\text{flagged}\;\;\text{at}\;\;\text{least}\;\;\text{once}}{100};$$

(10) $$\text{Sensitivity}=\frac{\text{number}\;\;\text{of}\;\;\text{alarms}\;\text{among}\;`\text{spiked}\;\;\text{outbreak}s^{\prime }\;\;\text{days}}{\text{number}\;\text{of}\;\text{outbreak}\;\text{days}};$$

(11) $$\text{Specificity}=\frac{\text{number}\;\;\text{of}\;\;\text{non}-\text{alarms}\;\;\text{among}\;\;\text{non}-`\text{spiked}\;\;\text{outbreak}s^{\prime }\;\;\text{days}}{\text{number}\;\text{of}\;\text{non}-\text{outbreak}\;\text{days}};$$

(12) $$\text{PPV}=\frac{\text{number}\;\text{of}\;\text{true}\;\text{positives}}{\text{number}\;\text{of}\;\text{positives}};$$

(13) $$\text{Timeliness}=\{\begin{array}{c} \frac{\sum_{\text{sim}=1}^{100}\left(`\text{spiked}\;\text{outbrea}k^{\prime }\;\text{detection}-1)/(\text{total}\;`\text{spiked}\;\;\text{outbrea}k^{\prime }\;\text{days}\right)}{100}\\ \;\;\;\text{if}\;\text{the}\;`\text{spiked}\;\text{outbrea}k^{\prime }\;\text{was}\;\text{detected}\\ 1\;\;\;\text{if}\;\text{the}\;`\text{spiked}\;\text{outbrea}k^{\prime }\;\text{was}\;\text{not}\;\text{detected}. \end{array}$$

A number of additional measures, such as receiver operator characteristic curves, have been used elsewhere to assess the performance of algorithms. However, here we focused on measures that can be easily explained to the users and policy makers who will be choosing which algorithms to implement. Hence, we have POD to measure the probability of detecting specific outbreaks but also specificity and sensitivity measures that tell users how accurate daily alarms will be during and outside outbreaks. PPV is useful when outbreaks are very rare because even if specificity is high, an alarm is more likely to be false than true. For example, a system monitoring two outbreak types (one from a common disease, and one from a rare disease) may have the same specificity for both of them; however, the PPV for the common disease would be higher than that of the rare disease. It is noted that in our study PPV is not very informative because we specified outbreak occurrence to be exactly one outbreak for each 343 day simulation. Thus, PPV does not give the user any more useful information than does specificity.

## 5 Simulation study results

As well as giving overall performance we report on differences between the 16 scenarios of synthetic syndromes. RAMMIE without trend, RAMMIE with trend, Farrington Flexible, EARS-C1, EARS-C2, EARS-C3 and EARS-NB were implemented to the most recent 49 weeks of each of the 100 simulations from each of the 16 signals (i.e. 49 weeks × 16 signals × 100 simulations = 78 400 simulated time series) and the evaluation measures defined in Section 4 were computed across all four sizes of ‘spiked outbreaks’ (notice that PPV is discussed at the end of this section). As previously mentioned, both RAMMIE versions were run on daily counts whereas Farrington Flexible and the EARS variants were run on 7-day moving totals. Results for RAMMIE with or without trend are very similar in cases where a trend does not exist; however, RAMMIE without trend produces a much lower specificity in some cases where data has an increasing trend, such as signal 13. Given that RAMMIE with trend produces a much higher specificity and similar sensitivity and timeliness (for signals with trend) as well as comparable results for signals without trend, we only include the results corresponding to RAMMIE with trend (referred to as RAMMIE) in the analysis below.

**Fig. 2.**
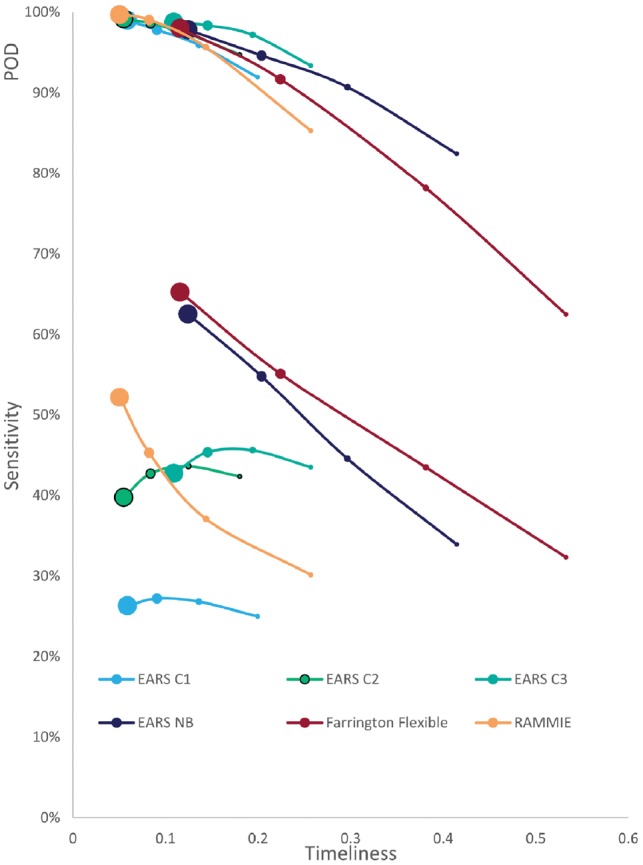
Average (across the 16 signals) sensitivity (lower end of plot) and POD (upper end of plot) versus timeliness for evaluating the impact of ‘spiked outbreak’ size on detection capabilities obtained from applying RAMMIE, Farrington Flexible, EARS-C1, EARS-C2, EARS-C3 and EARS-NB to the most recent 49 weeks of each of the 100 simulations of the 16 signals. Marker size is proportional to outbreak size (i.e. largest point refers to large outbreaks; second largest refers to medium outbreaks; third largest refers to small outbreaks; smallest point refers to very small outbreaks)

We first investigate how performance is affected by outbreak size. Figure 2 shows the detection capability of each algorithm for different outbreak sizes. It displays both POD and sensitivity versus timeliness (note that a lower score for timeliness indicates better results). Specificity was not included in this figure because it varies only very slightly with outbreak size (for each of the algorithms, a difference of less than 0.003, on average, between the different outbreak sizes). The average specificity across all 16 signals and all outbreak sizes for each of the algorithms is: 0.981 for Farrington Flexible; 0.953 for RAMMIE; 0.922 for EARS-C1; 0.834 for EARS-C2; 0.812 for EARS-C3; 0.969 for EARS-NB. The figure shows that, all algorithms were very likely to detect the larger outbreaks, but POD was considerably lower for the smallest outbreaks, particularly for the Farrington Flexible method. Farrington Flexible detection capability is the most affected by outbreak size, though generally algorithm ranking is not affected by outbreak size and as the latter increases, POD, sensitivity and timeliness improve. Farrington Flexible and EARS-NB have a much higher sensitivity than RAMMIE but lower POD, most likely due to the smoothing methods used in adjusting from weekly to daily surveillance. In particular, Farrington Flexible has the highest sensitivity but lowest POD, though its POD is similar to the other algorithms with outbreaks of size 10. EARS-C1 and EARS-C2 were the most timely, although RAMMIE has similar timeliness except for the smallest outbreaks. (See Supplementary Fig. S4 for further demonstration on how detection is influenced by outbreak size).

**Fig. 3.**
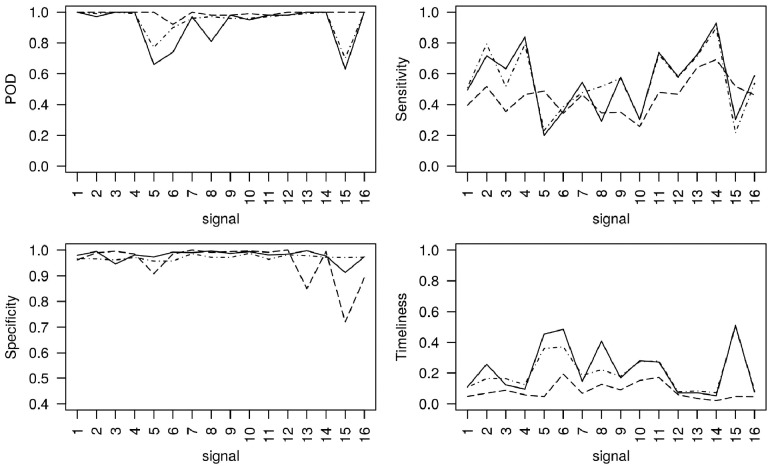
POD, sensitivity, specificity and timeliness for each of the simulated signals, with added medium ‘spiked outbreaks’, obtained from applying RAMMIE (dashed lines), Farrington Flexible (solid lines) and EARS-NB (dot dash lines) to the most recent 49 weeks of each of the 100 simulations from each signal

Second, we investigate the algorithms’ performance with the particular characteristics depicted by each of the 16 simulated signals. Figure 3 displays the algorithms’ 4 performance measures for each of the 16 signals, when ‘spiked outbreaks’ of medium size (*m *=* *5) were added to the most recent 49 weeks. Figures corresponding to very small, small and large outbreaks can be found in the Supplementary Data. EARS-C1, EARS-C2 and EARS-C3 have much lower sensitivity and specificity than the other algorithms making them not very useful in our setting, therefore Figure 3 reports on the results corresponding to just RAMMIE, Farrington Flexible and EARS-NB. The figure shows that Farrington Flexible has the highest specificity on average followed by EARS-NB then RAMMIE. RAMMIE specificity is similar to Farrington Flexible, however it has particularly low values for signals 5, 13 and 15, all of which have high volume and seasonality. Signal 13 has a trend and signals 5 and 15 have the added ‘seasonal outbreaks’, which could explain the low specificity. RAMMIE produces particularly low specificity for signal 15 and so the false alarms might be due to the fact that RAMMIE detects ‘seasonal outbreak’ better (which in reality could be the seasonal influenza outbreak). RAMMIE sensitivity is the least variable across signals. Farrington Flexible produces low POD and is the least timely particularly for signals 5, 6, 8 and 15 which have high seasonality and added seasonal outbreaks (for signals 5, 6 and 15). EARS-NB gives a similar picture but with a higher POD and lower timeliness on average. The timeliness for simulations where an outbreak is not detected is set to 1, which explains why signals with particularly low POD are also the least timely. RAMMIE produces, on average, the highest (lowest) and most consistent POD and timeliness across all signals.

Every algorithm scored highly in terms of PPV. Farrington Flexible had the highest overall PPV (99.73%) followed by EARS-NB (99.56%), RAMMIE (99.04%), EARS-C1 (99.03%), EARS-C2 (98.00%) and EARS-C3 (97.70%). The slightly lower values for EARS-C methods reflects their lower scores for specificity.

## 6 Discussion

During the testing of algorithm performance across a range of scenarios, we found that EARS-C1, EARS-C2 and EARS-C3 have considerably lower specificity than the other algorithm variants tested. Amongst the other algorithms, Farrington Flexible has the highest sensitivity and specificity, whereas RAMMIE has the highest POD and is the most timely.

Farrington Flexible and EARS-NB smooth the data by taking moving totals and so dilute the signal, whereas RAMMIE is designed for daily surveillance which allows it to detect more outbreaks, typically 2–3 days earlier. However, RAMMIE is less consistent in generating alarms during spiked outbreak days and produces the lowest sensitivity. Due to smoothing of the day-of-the-week effects, once Farrington Flexible and EARS-NB detect an outbreak, they are more likely to generate alarms consistently during the remaining spike outbreak days.

Although there are differences in the performance measures across the syndromes, the differences mostly affect all the algorithms in similar ways, apart from some signals with high seasonality or added seasonal outbreaks. The Flexible Farrington method has the biggest variation in detection capabilities across the different signals. Our results show that the performance of RAMMIE can be improved by adjusting it for long-term trends.

### 6.1 Implications for public health authorities

In this paper, we provide an assessment of algorithm detection capability to help decision makers and researchers performing daily surveillance decide which algorithm would be more efficient for their needs, and which aspects of detection are more important i.e. POD, sensitivity, specificity, PPV or timeliness. POD is the most important measure if the priority of the surveillance system is to ensure that all outbreaks are detected. Whilst our sensitivity measure is important if a clear consistent signal is required, i.e. an alarm every day during an outbreak, specificity is important if the user needs to ensure that there are no false alarms. PPV provides a measure of how likely an alarm is to be true, which is particularly important when outbreaks do not occur often. Finally, timeliness may be the most important measure if the focus of the surveillance activity is on providing early warning and other public health systems exist which can reliably detect outbreaks. For each of these measures the user may want to set a minimum threshold required by the algorithms and/or prioritize which measures are most important for their surveillance needs. Furthermore, their requirements may vary depending on the public health hazard they are trying to detect.

In effect, there is no one algorithm that is better across all detection measures. However, due to their lower specificity, the EARS-C1, EARS-C2 and EARS-C3 variants may not be as suited for a multi-purpose daily surveillance system. Farrington Flexible had the highest sensitivity and specificity so may be preferred if the priority is for daily alarms that are as accurate as possible. However, RAMMIE was more timely and had a slightly higher POD so it may be more useful where early warning is important or the top priority is that at least one alarm occurs during an outbreak. Alternatively, EARS-NB may be preferred as a compromise because its sensitivity was better than RAMMIE and its POD slightly better than Flexible Farrington.

Specifically, we provide PHE with an evaluation of RAMMIE that will help them improve their service by modifying RAMMIE or replacing it with another algorithm. We recommend adjusting RAMMIE to allow for any long-term trends in the underlying syndromic data. We also provide a range of developed simulations that researchers can use in testing other algorithms for use in a daily setting elsewhere (We aim in the future to provide public access to these simulations).

One of our research aims was to discover which algorithm worked best in particular situations, (e.g. which algorithm is best for small numbers or which is best for 5-day-week systems, or which is best for different outbreak sizes or types). However, we show that the ranking of algorithms is not affected by these different situations. Therefore, whichever algorithm is preferred by users should be used for all types of signals.

### 6.2 Limitations

The timeliness penalty of 1 for failing to detect an outbreak is set quite high. Consequently, when an algorithm has a low POD score it will also have very poor timeliness, e.g. Farrington Flexible for signals with seasonal outbreaks. An alternative approach, given that the simulated outbreaks are nearly symmetric could be to impose a penalty of 0.5, because it is highly unlikely that an outbreak will first be detected after it has peaked. It would also be possible to not impose any penalty, although that could result in algorithms that can only detect big outbreaks with an initial sharp rise in cases being scored as more timely than algorithms with a much better POD.

### 6.3 Future work

In further research, we aim to explore different decision rules for evaluating the algorithms. We can use the results from this study to see how different priorities for timeliness, sensitivity, specificity or POD would affect the decision of which algorithm we should use. For instance, we can ask decision makers to specify a set of preferred requirements for algorithms (e.g. specificity > 98%) and their priorities, (e.g. whether timeliness is a priority over alarming every day). Then we can apply these decision rules to our study results to determine which algorithm performed best against the criteria set by the decision makers. This approach would also allow them to set different priorities for different public health events (e.g. a short spike in vomiting cases caused by a norovirus outbreak or a longer-term gradual rise in scarlet fever incidence).

We attribute some of the differences in performance between algorithms to whether or not they were designed for daily or weekly surveillance. Future work could create new versions of the Farrington Flexible and EARS algorithms which are specifically adapted to model day-of-the-week effects inherent in the daily surveillance data.

This research focuses on the detection of spiked outbreaks. The seasonal outbreak detection is part of the bigger question of what are we trying to model versus what we are trying to detect. This is an issue that can be addressed in future work. Also, further research on RAMMIE day-of-the-week detection efficiency (e.g. weekends versus weekdays; public holidays versus non-public holidays; detection on different days of the week) can be undertaken.

## 7 Conclusion

We have created simulated data representing the wide range of data structures seen in a multi-purpose daily surveillance system. We have used this data to compare three algorithms already in use and made these modelled data structures available for the evaluation of other new algorithms. We have shown that the decision as to which algorithm to use should depend on which detection characteristics are most important to the user and not the characteristics of the data signal being monitored. In particular, the Farrington Flexible method has the highest sensitivity and specificity, whereas RAMMIE has the highest POD and is the most timely.

## Supplementary Material

bty997_Supplementary_Data
