# Supplementary material for: Comparison of statistical algorithms for daily syndromic surveillance aberration detection
Source: Bioinformatics. 2019 Jan 25;35(17):3110–8. doi: 10.1093/bioinformatics/bty997 (PMC6736430; doi:10.1093/bioinformatics/bty997)
Supplement: bty997_Supplementary_Data [file bty997_supplementary_data.pdf]

# Supplementary Information

### Evaluating statistical aberration detection algorithms for multi-purpose daily syndromic surveillance.

Angela Noufaily[1], Roger A. Morbey[2,6], Felipe J Colón-González[3,6], Alex J. Elliot[2,6], Gillian E. Smith[2,6], Iain R. Lake[3,5,6], Noel McCarthy[4,5]

[1] Statistics and Epidemiology, Warwick Medical School, University of Warwick, Coventry, CV4 7AL, UK.
[2] National Infection Service, Public Health England, 5 St Philip's Place, Birmingham, B3 2PW, UK.
[3] School of Environmental Sciences, University of East Anglia, Norwich, NR4 7TJ, UK.
[4] Population Evidence and Technologies, Warwick Medical School, University of Warwick, Coventry, CV4 7AL, UK.
[5] NIHR Health Protection Research Unit in Gastrointestinal Infections, University of Liverpool, UK.
[6] NIHR Health Protection Research Unit in Emergency Preparedness and Response, Kings College London, London, UK.

## Supplementary data

### Public Holidays

Public holiday effects are added to the data following the addition of 'seasonal outbreaks' and 'spiked outbreaks'. To add the public holidays in the most realistic way, the simulated 7-year data are considered like years 2010-2016, with a difference that our simulated data consists of years with equal number of days (364 days each) - the month of July consists of just 30 days rather than 31 and February includes 28 days always. This means that each year starts on Monday, January 1$^{st}$ and ends on Sunday, December 31$^{s}t$. Public holiday dates are then set in a very similar way to the years 2010-2016 in England, as shown in Table 1. As for the public holiday effects: in the 5 day system, the public holiday count was set to zero and the weekday after the public holiday was multiplied by 1.5; in the 7 day system, the bank holiday count was doubled.

Table 1: Bank holiday dates.

| | Year | | | | | | |
|---|---|---|---|---|---|---|---|
| | 2010 | 2011 | 2012 | 2013 | 2014 | 2015 | 2016 |
| Jan | 1 | 1 | 1 | 1 | 1 | 1 | 1 |
| Feb | - | - | - | - | - | - | |
| Mar | - | - | - | 30 | - | - | - |
| Apr | 6, 9 | 20, 23, 27 | 6, 9 | 2 | 20, 23 | 6, 9 | 13, 16 |
| May | 7, 28 | 7, 28 | 7 | 7, 28 | 7, 28 | 7, 28 | 7, 28 |
| Jun | - | - | 4, 5 | - | - | - | - |
| Jul | - | - | - | - | - | - | - |
| Aug | 28 | 28 | 28 | 28 | 28 | 28 | 28 |
| Sep | - | - | - | - | - | - | - |
| Oct | - | - | - | - | - | - | - |
| Nov | - | - | - | - | - | - | - |
| Dec | 25, 26 | 25, 26 | 25, 26 | 25, 26 | 25, 26 | 25, 26 | 25, 26 |

## Figures

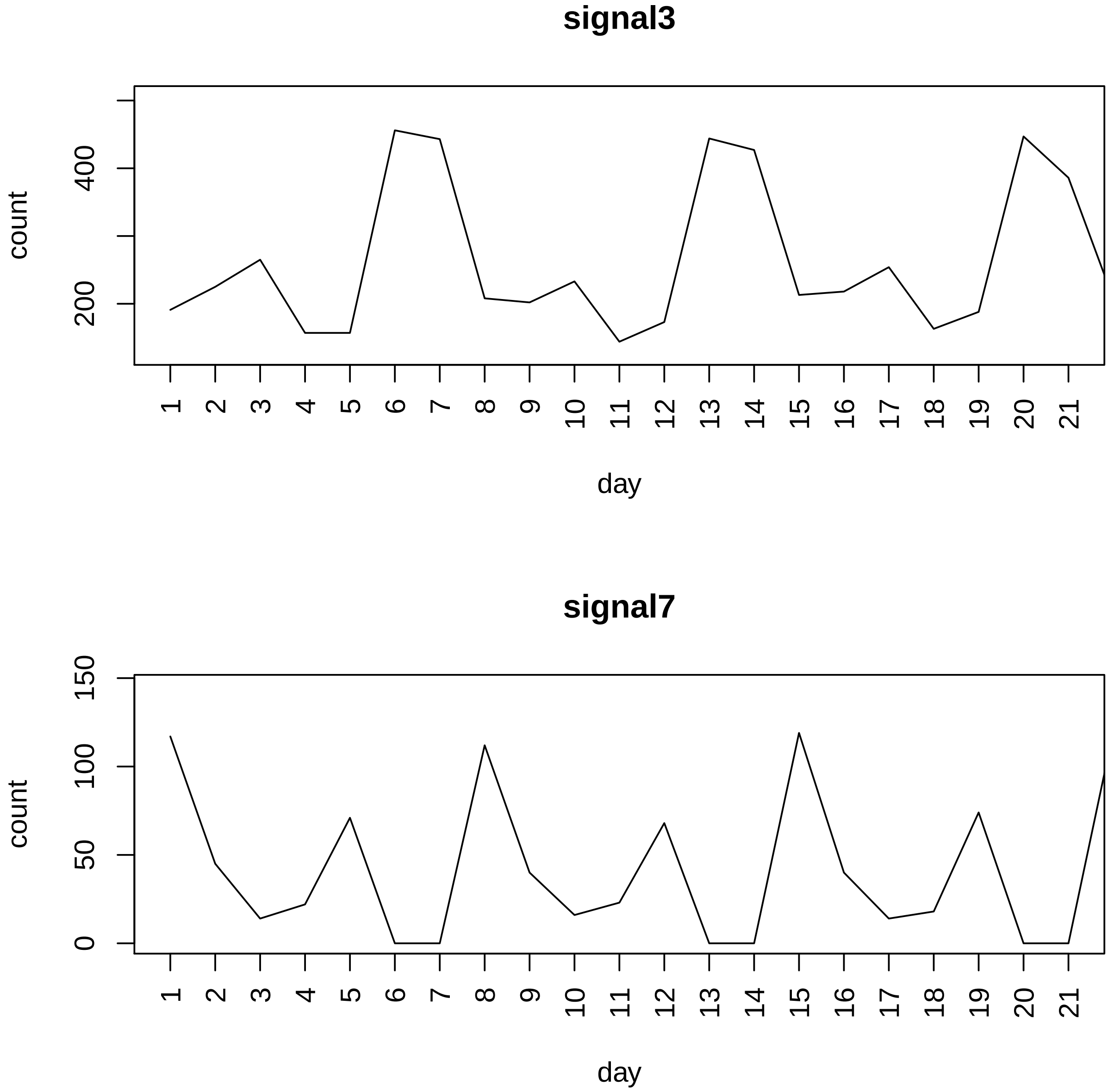


Figure 1: Weekly patterns for signals 3 and 7; the first 3 weeks are shown.

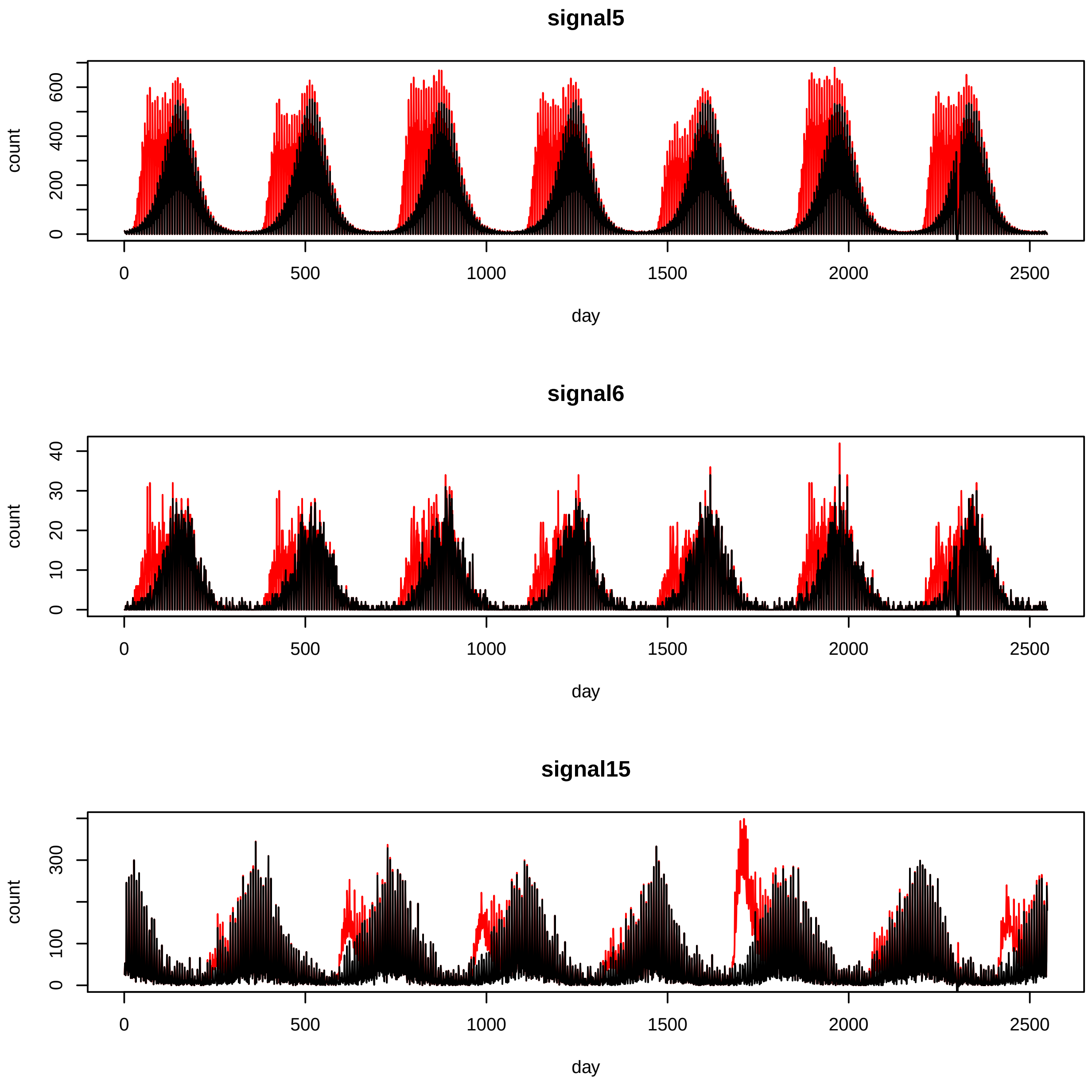


Figure 2: Plots of signals 5, 6 and 15 with added (in red) 'seasonal outbreaks'.

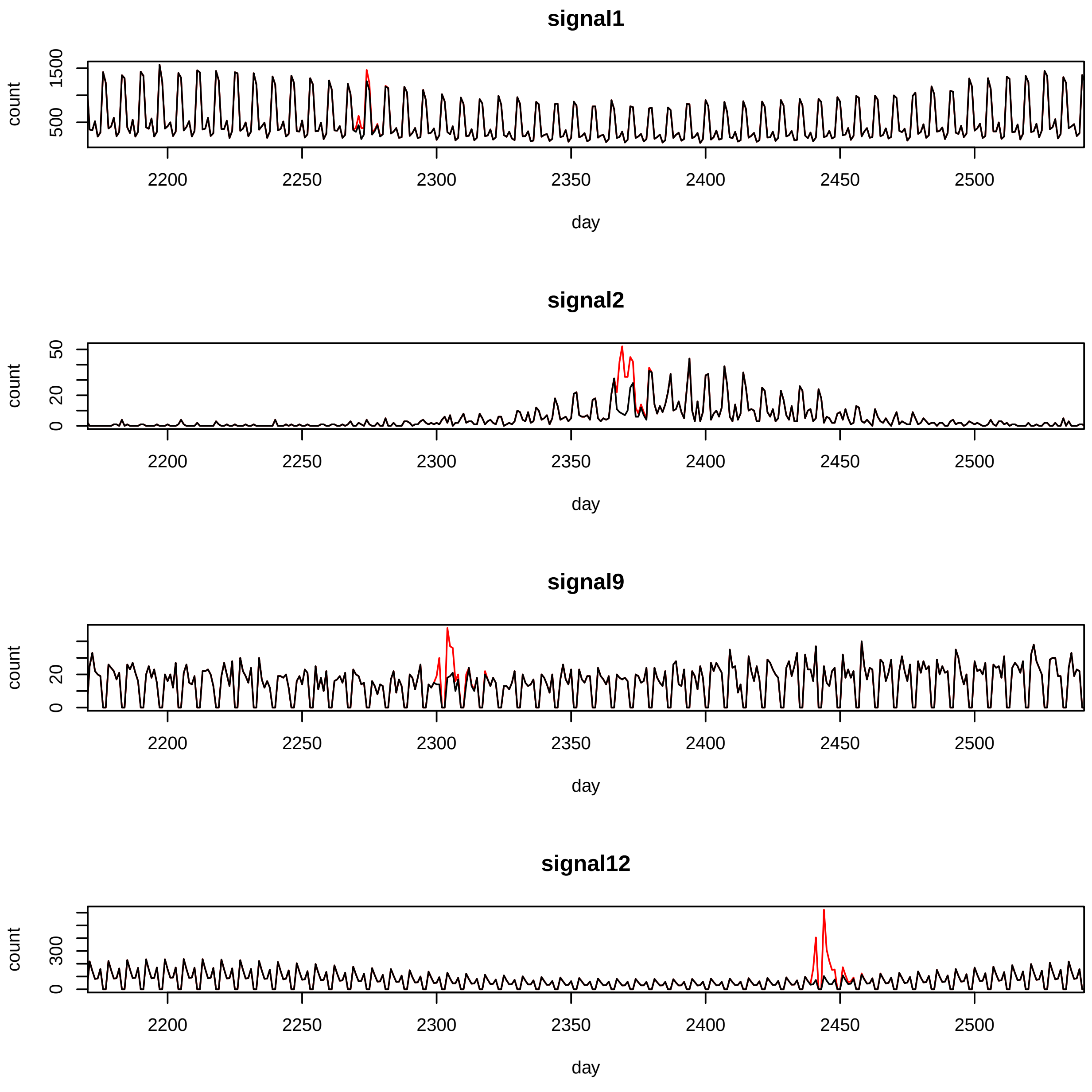


Figure 3: Plots of the most recent 49 weeks of signals 1, 2, 9 and 12 with added (in red) medium 'spiked outbreaks'.

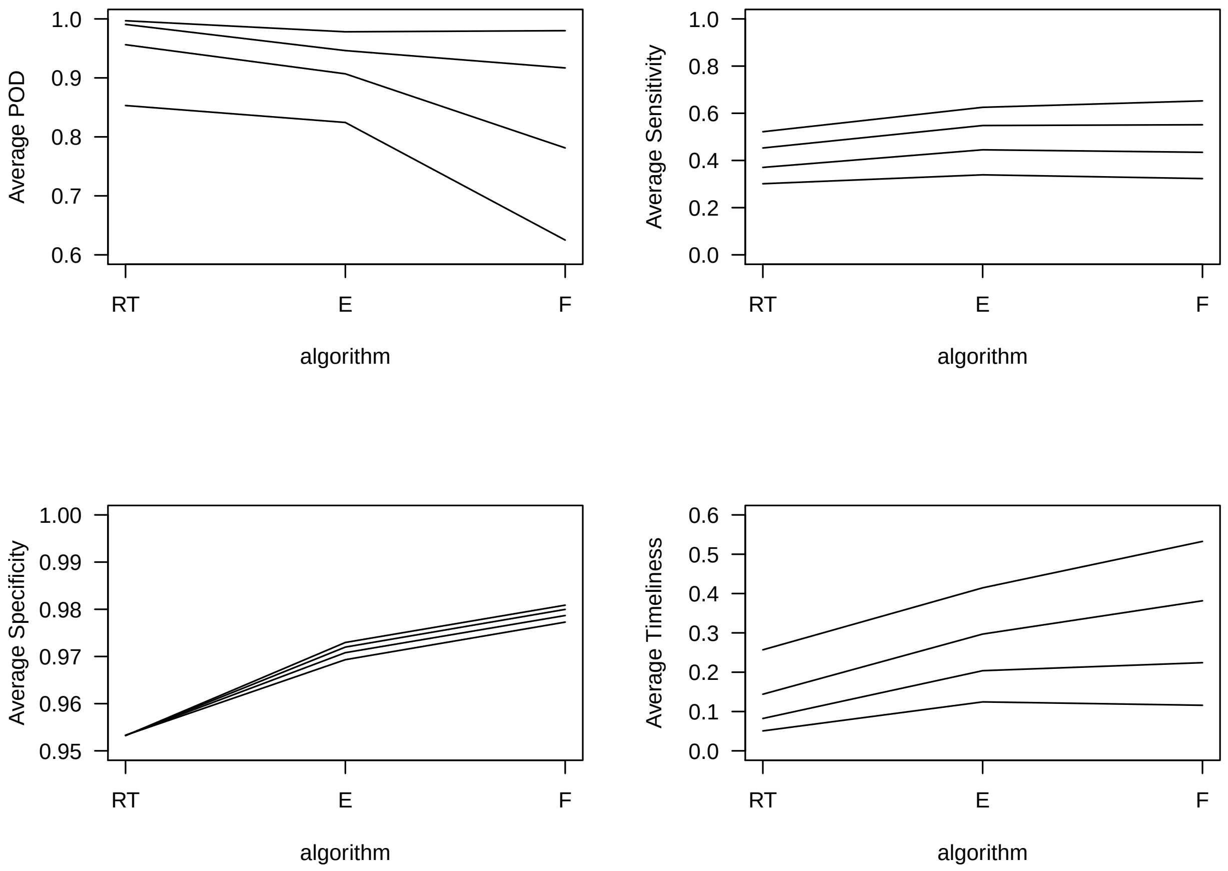


Figure 4: Average POD, sensitivity, specificity and timeliness over the 16 signals when ‘spiked outbreaks’ of sizes: very small, small, medium and large (lower curve to higher respectively) are added to the most recent 49 weeks.

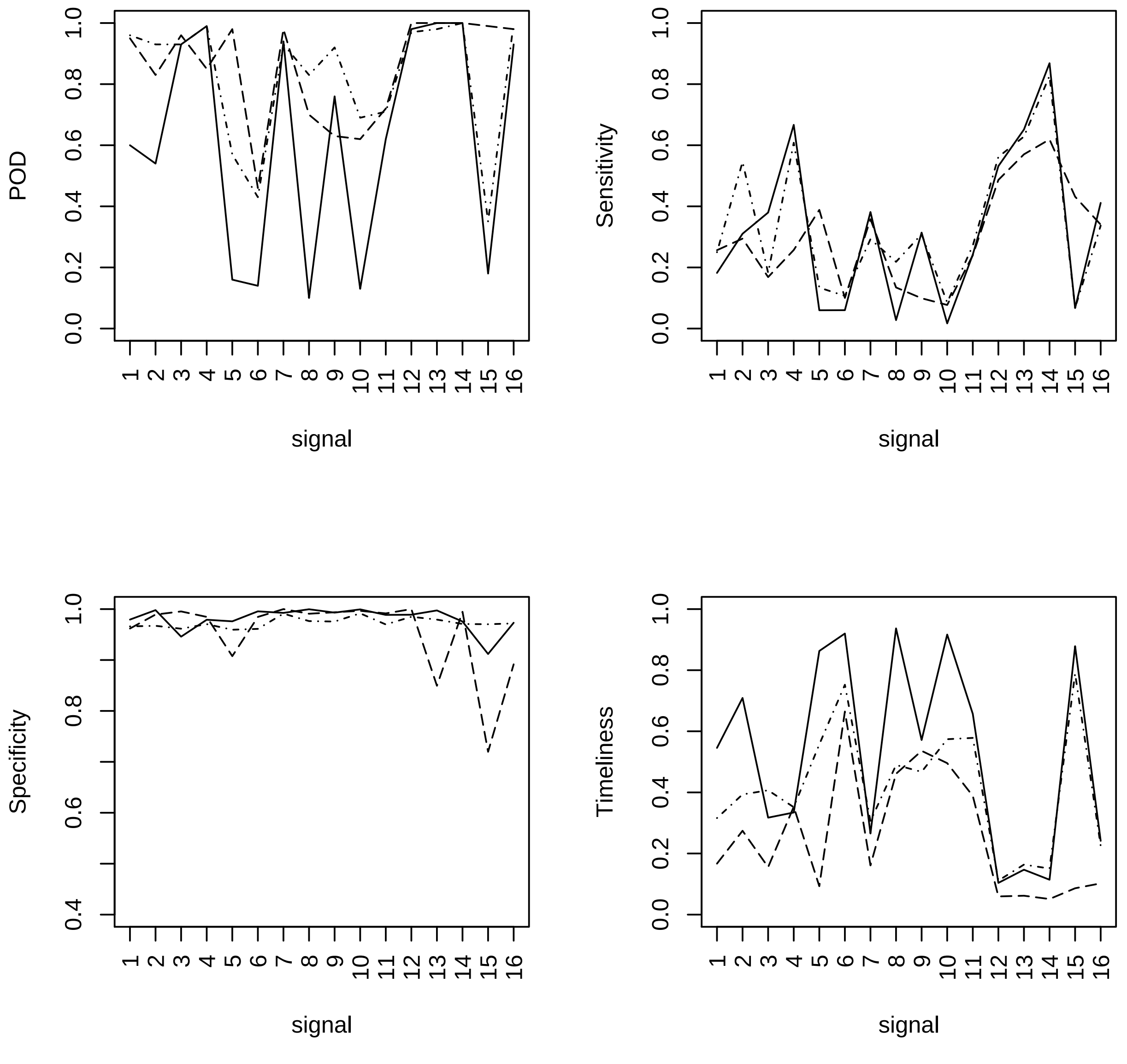


Figure 5: POD, sensitivity, specificity and timeliness for each of the simulated signals, with added very small 'spiked outbreaks', obtained from applying RAMMIE (dashed lines), Farrington Flexible (solid lines) and EARS-NB (dot dash lines) to the most recent 49 weeks of each of the 100 simulations from each signal.

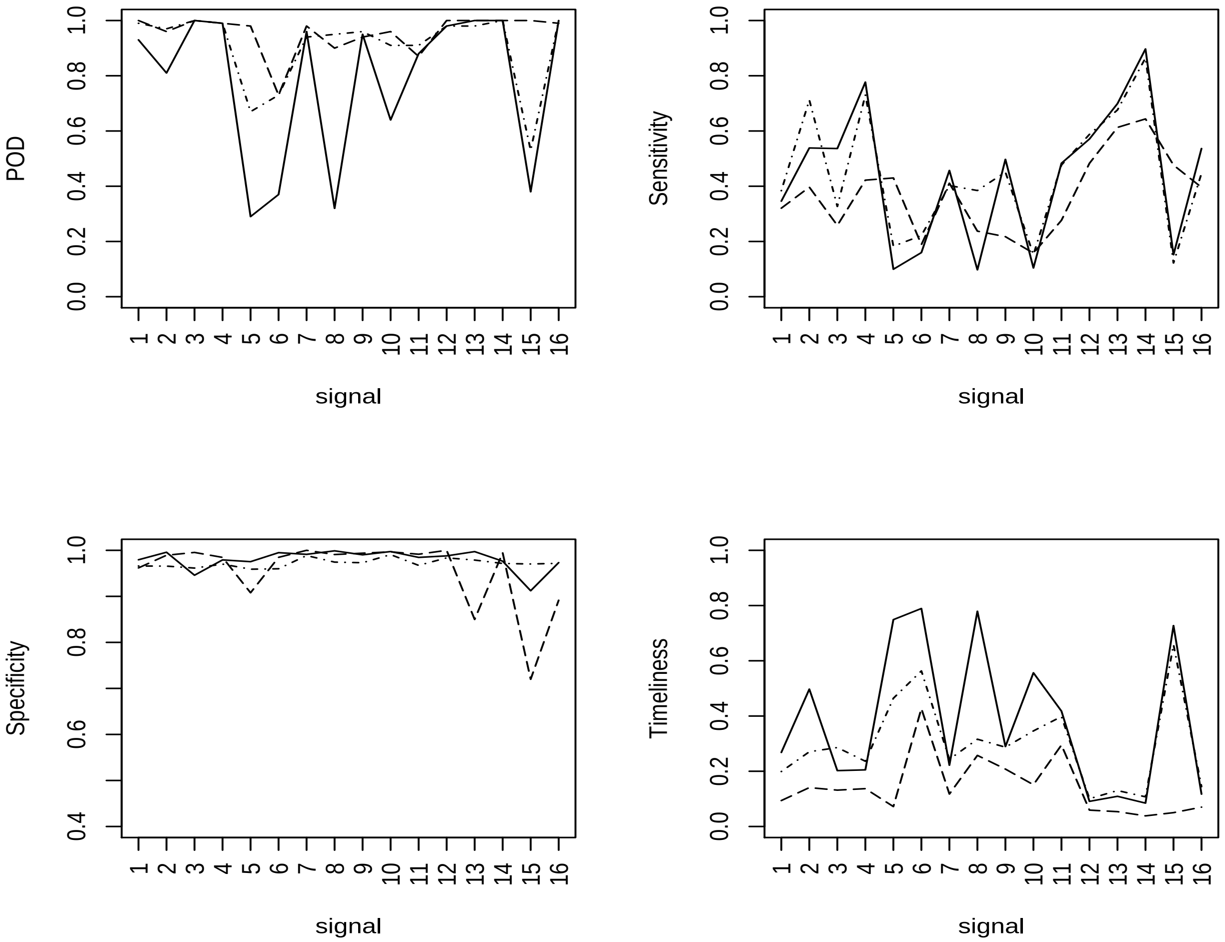


Figure 6: POD, sensitivity, specificity and timeliness for each of the simulated signals, with added small 'spiked outbreaks', obtained from applying RAMMIE (dashed lines), Farrington Flexible (solid lines) and EARS-NB (dot dash lines) to the most recent 49 weeks of each of the 100 simulations from each signal.

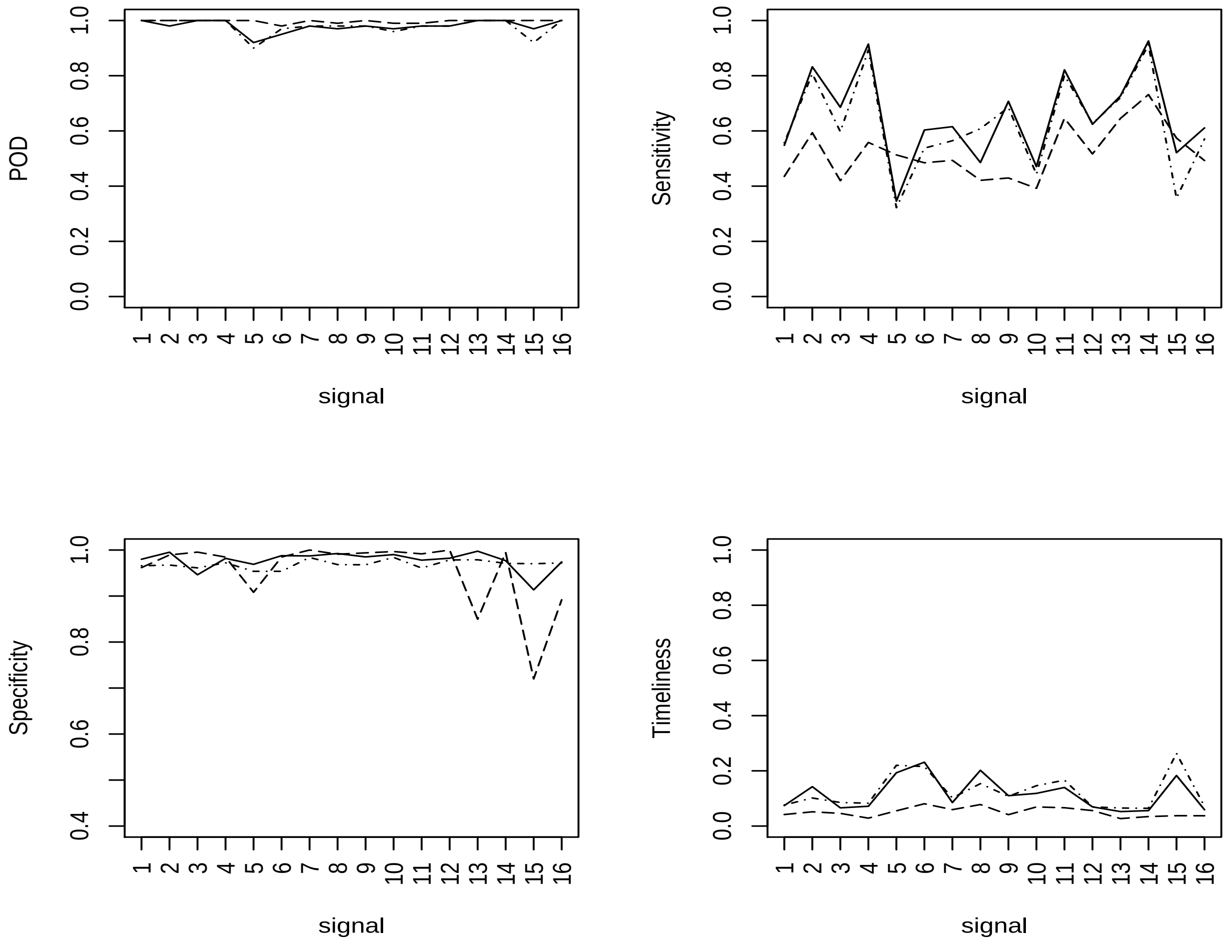


Figure 7: POD, sensitivity, specificity and timeliness for each of the simulated signals, with added large 'spiked outbreaks', obtained from applying RAMMIE (dashed lines), Farrington Flexible (solid lines) and EARS-NB (dot dash lines) to the most recent 49 weeks of each of the 100 simulations from each signal.
